# *PLAG1* fusions extend the spectrum of PLAG(L)-altered CNS tumors

**DOI:** 10.1007/s00401-023-02643-4

**Published:** 2023-10-23

**Authors:** Arnault Tauziède-Espariat, Aurore Siegfried, Yvan Nicaise, Delphine Dghayem, Anne Laprie, Vincent Lubrano, Pomone Richard, Guillaume Gauchotte, Joséphine Malczuk, Olivier Klein, Lauren Hasty, Alice Métais, Fabrice Chrétien, Volodia Dangouloff-Ros, Nathalie Boddaert, Felix Sahm, Philipp Sievers, Pascale Varlet, Emmanuelle Uro-Coste, the RENOCLIP-LOC

**Affiliations:** 1 Department of Neuropathology, GHU Paris-Psychiatrie et Neurosciences, Sainte-Anne Hospital, 1, Rue Cabanis, 75014 Paris, France; 2 Department of Pathology, Toulouse University Hospital, Toulouse, France; 3 INSERM U1037, Cancer Research Center of Toulouse (CRCT), Toulouse, France; 4 Université Paul Sabatier, Toulouse III, Toulouse, France; 5 Department of Radiology, Purpan University Hospital, Toulouse, France; 6 Department of Medical Oncology, IUCT-Oncopole, Toulouse, France; 7 Department of Neurosurgery, Clinique de l’Union, Toulouse, France; 8 Department of Pathology, Medipath Laboratory, Toulouse, France; 9 Department of Pathology, CHRU, Nancy, France; 10 Department of Pediatric Neurosurgery, EA 3450, DeVAH (Développement Adaptation et Handicap), CHRU Nancy et Universite de Lorraine, Nancy, France; 11 Université Paris Cité, Institute of Psychiatry and Neuroscience of Paris (IPNP), INSERM U1266, Ima-Brain Team, 75014 Paris, France; 12 Paediatric Radiology Department, Hôpital Necker Enfants Malades, AP-HP, University de Paris, INSERM U1163, Institut Imagine, Paris, France; 13 Department of Neuropathology, Institute of Pathology, University Hospital Heidelberg, Heidelberg, Germany; 14 Clinical Cooperation Unit Neuropathology, German Consortium for Translational Cancer Research (DKTK), German Cancer Research Center DKFZ), Heidelberg, Germany

Central Nervous System (CNS) embryonal tumors with PLAG-family amplification have been isolated by a distinct DNA-methylation profile [1]. These tumors are characterized by recurrent *PLAGL1* or *PLAGL2* amplifications [1, 4]. In some cases, no amplification of these genes was found (9.7%, 3/31) [1]. Here, we report two cases, classified as being part of the “embryonal tumor with PLAG-family amplification” methylation class (MC), that did not have a PLAG-family amplification but instead harbored a *PLAG1* fusion*.* The aim of our work was to compare the clinical, radiological and histopathological features of these cases with previously published cases having a *PLAGL1/2* amplification.

**Fig. 1:**
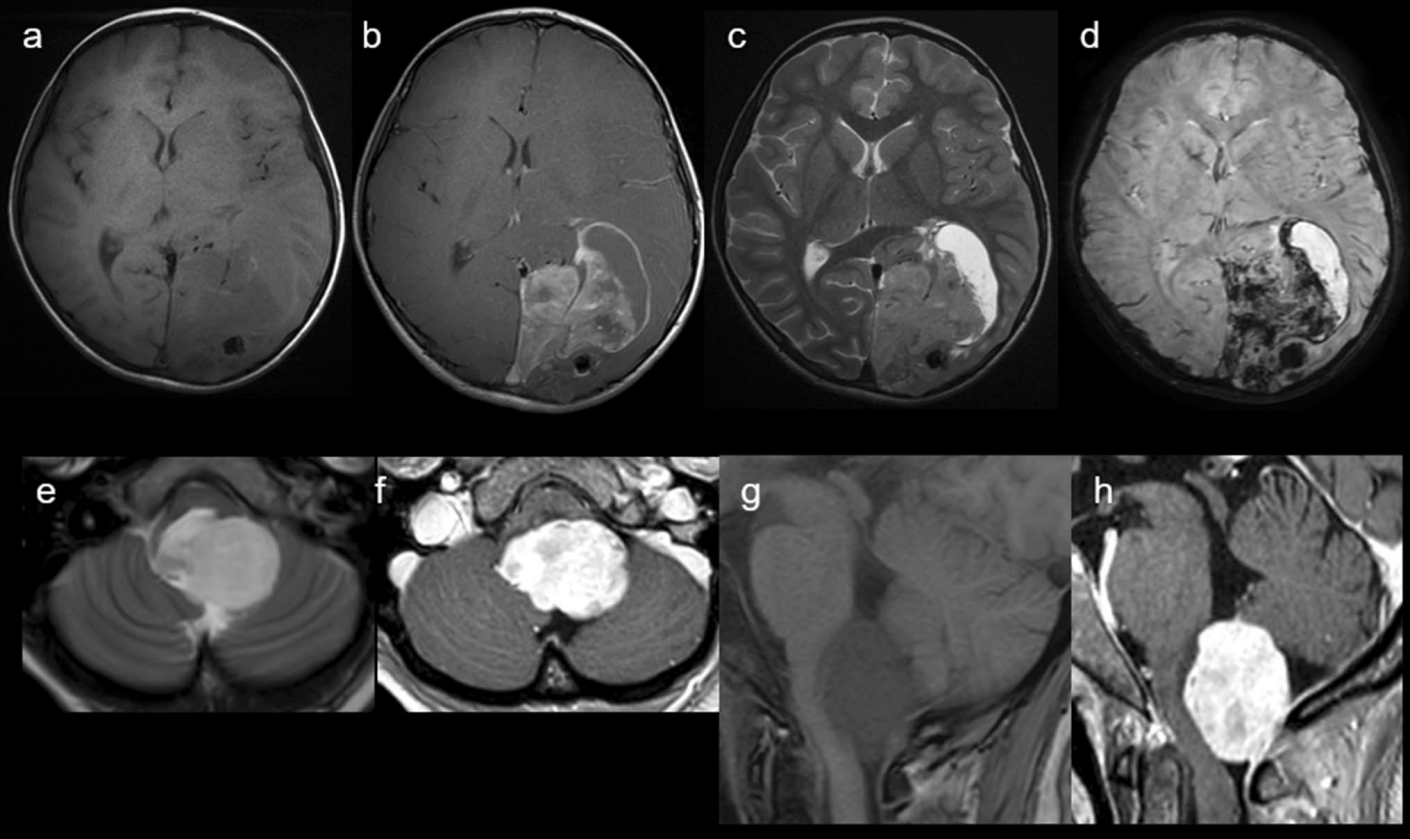
Radiological features of #cases 1 and 2. Case#1 (**a**–**d**): **a** MR images show a left occipital mass with tissue and cysts, low signal intensity on T1-weighted images, and strong contrast enhancement (**b**), heterogeneous high signal intensity on T2-weighted images (**c**) and extended hemorrhage on susceptibility-weighted images (**d**). Case#2 (**e**–**h**): **e**–**g** MR images show a mass dorsally exophytic from the medulla oblongata, with high signal intensity on T2-weigthed images, low signal intensity on T1-weigthed images (**f**–**h**) and very strong and homogeneous contrast enhancement. *MR* magnetic resonance

**Fig. 2:**
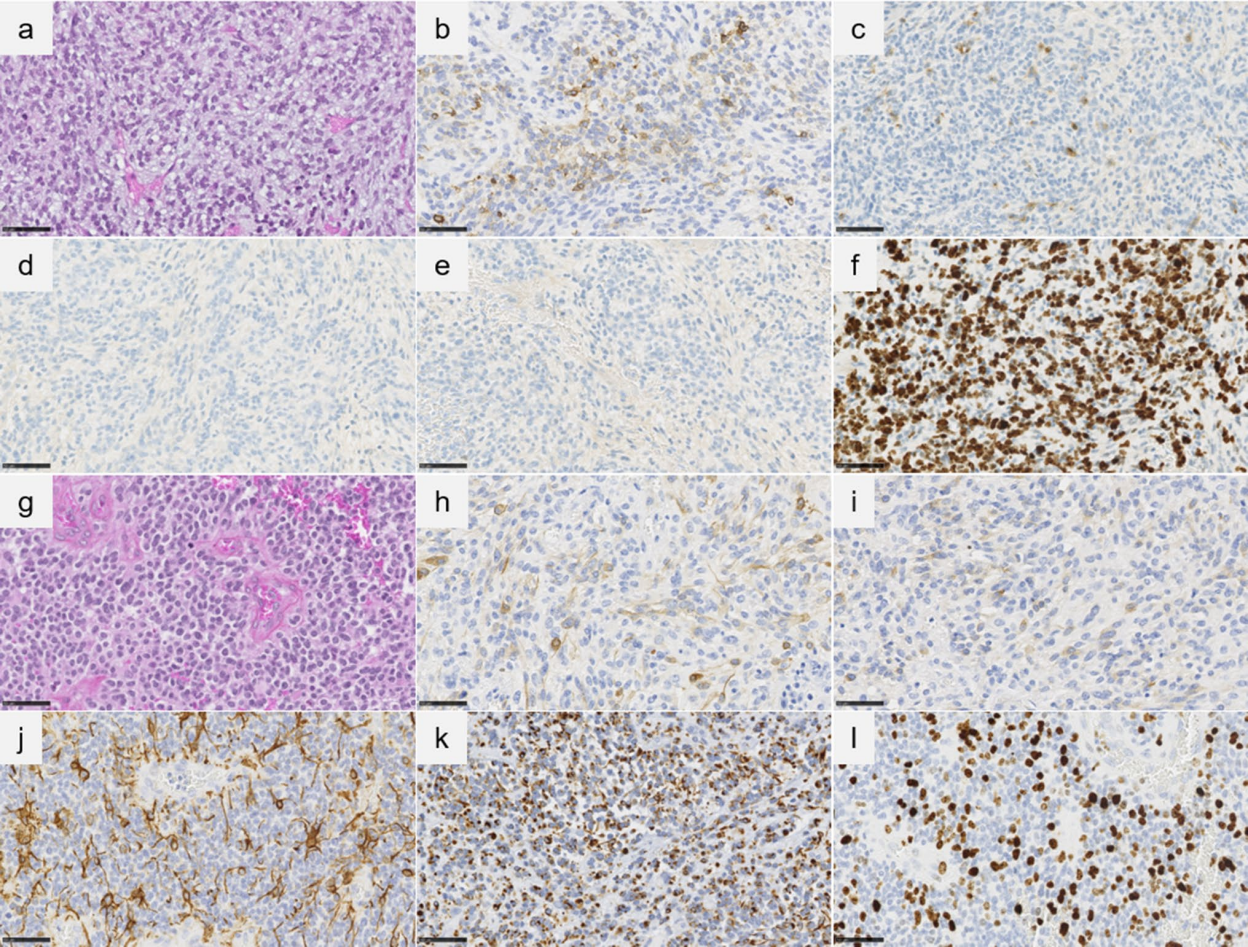
Histopathological features of #cases 1 and 2. Case#1 (**a**–**f**): **a** Neoplasm composed of monotonous round tumor cells with microcystic modifications (HPS, magnification 400×). **b–c** A subset of tumor cells express neuronal markers (synaptophysin and MAP2, magnification 400×). **d**–**e** No expression of glial markers (GFAP and Olig2, magnification 400×). **f** High MIB labeling index (magnification 400×). Case#2 (**g**–**l**): **g** Neoplasm composed of monotonous round tumor cells with mitoses and microvascular proliferation (HPS, magnification 400×). **h**, **i** A subset of tumor cells expressing neuronal markers (synaptophysin and MAP2, magnification 400×). **j** Residual expression of GFAP by staining of scattered tumor cells (magnification 400×). **k** Expression of desmin (magnification 400×). **l** High MIB labeling index (magnification 400×). *HPS* hematoxylin phloxin and saffron. Black scale bars represent 50 μm

The two observations concerned a 6-year-old boy (Case #1) and a 39-year-old woman (Case #2). The tumors were located in the left occipital lobe (Case #1) (Fig. 1a–d) and in the fourth ventricle with another location in the left temporal lobe and a leptomeningeal dissemination (Case #2) (Fig. 1e–h). Neuroradiological review revealed large tissular and cystic tumors, having strong enhancement after contrast injection (Fig. 1). Histopathological review revealed that both cases presented similar features (Fig. 2). They were well circumscribed from the brain/cerebellar parenchyma and composed of sheets of monotonous oval cells with round to oval nuclei and a pale cytoplasm (Fig. 2a and g). In some areas, an epithelioid pattern with sharply demarcated tumor cells was present. A dense branching capillary network (with microvascular proliferation in case #2) was observed. No rosettes, rhabdoid component or pseudorosettes were discovered. Hemorrhagic and microcystic modifications were present. Necrosis was absent, but the mitotic count and proliferation index were high (Fig. 2f and l). Immunohistochemistry detected a preserved expression of INI1, BRG1 in the tumor cells but there was no immunopositivity for LIN28A or BCOR. Neuronal markers (MAP2 and synaptophysin) were constantly expressed (Fig. 2b, c and h, i), whereas there was no or only focal expression of glial markers (Fig. 2d, e and j). Desmin was expressed in case #2 (without expression of smooth muscle actin or myogenin) (Fig. 2k). All of these results resembled the reported CNS embryonal tumors with PLAG-family amplification [1, 4]. Using the Heidelberg DNA-methylation classifier (v12.5), case #1 was classified as a CNS embryonal tumor with PLAG-family amplification (having a calibrated max-score of 0.99), whereas the second case did not present a significant calibrated score for a MC (despite good DNA integrity/quantity and performance of bisulfide conversion). They both clustered in vicinity of this MC by t-Distributed Stochastic Neighbor Embedding (t-SNE) analysis (Supplementary Fig. 1). RNA sequencing analysis of the two cases showed a fusion between *PLAG1* and *TCF4* (case #1) and *EWSR1* (case #2) genes (Supplementary Fig. 2). For Case #1, a gross total resection of the tumor was performed followed by craniospinal radiation therapy. A posterior fossa metastasis was discovered 8 months later and was treated by gross total resection, chemotherapy and radiation therapy. Histopathologically, the second lesion was similar to the primary tumor. The patient was alive without novel progression at the end of follow-up, 65 months after the initial disease. For Case #2, a gross total resection of the posterior fossa tumor was performed, followed by craniospinal radiation therapy. At the latest follow-up, 16 months after the first surgery, the patient was alive without recurrence in the posterior fossa and with a stable disease in the supratentorial area.

The novel embryonal tumor with amplification of the *PLAGL1/2* genes mainly concerns children (85% of reported cases, ranging from 0 to 36 years old) and may be located all along the neuraxis (mostly hemispheric but also infratentorial and ventricular) [1, 4]. The sex ratio female: male is 1.4 [1, 4]. Radiologically, few data are available. These tumors seem to be well-circumscribed, voluminous tissular and cystic masses that show strong enhancement after injection of gadolinium [4]. Histopathologically, they are considered embryonal tumors and present a pluriphenotypic immunoprofile (with an expression of neuronal markers, focal expression of glial markers, and a frequent staining for desmin but without any immunoreactivity for the other myogenic markers) [1, 4]. Because of their poorly differentiated morphology, initial diagnoses were variable: glioneuronal tumors, sarcomas, medulloblastomas or high-grade gliomas, according to the tumor location [1, 4]. The two current cases were in line with all these clinical, radiological and histopathological features. However, contrarily to the first description, they did not harbor *PLAGL1/2* amplifications but rather a fusion implicating the *PLAG1* gene. To date, in the CNS, PLAG-family gene alterations have been implicated in two different tumor types. *PLAGL1/2* amplifications have been reported in a subgroup of embryonal tumors, whereas *PLAGL1* fusions have been described in ependymoma-like neuroepithelial tumor (NET) [1, 3]. Fusions of *PLAG1,* a gene of the PLAG-family, have not been reported in the CNS. They have been reported in several tumors, such as salivary gland pleomorphic adenoma, lipoblastoma, and myoepithelial carcinoma [2]. To our knowledge, no fusions implicating the *TCF4* and *EWSR1* genes have been reported in these tumors. Cases of ependymoma-like NET with *PLAGL1* fusions [3] were distinct from ours in terms of location (all NET were supratentorial), histopathology (NET present frequent ependymal features) and immunohistochemistry (constant expression of GFAP in NET) [3]. Using t-SNE analysis, our cases clustered in close vicinity with the MC of embryonal tumors with *PLAGL1/2* amplifications. Three cases out of 31 (9.7%) of DNA-methylation based embryonal tumors with *PLAGL1/2* did not harbor any amplifications of these genes, and to our knowledge, *PLAG1* fusions were not explored [1]. Data concerning the outcome of patients with embryonal tumors with PLAG-family amplifications, seems to evidence a high rate of recurrences and a poorer prognosis in cases with *PLAGL2* amplifications [1, 4]. More reports are necessary to determine any potential benefit of chemotherapy and craniospinal irradiation in the treatment of these rare tumors.

To conclude, we report, for the first time, two embryonal tumors with *PLAG1* fusions sharing clinico-radiological, histopathological, immunohistochemical, and epigenetic similarities to CNS embryonal tumors with PLAG-family amplification. Consequently, *PLAG1* fusions expand the spectrum of the alterations encountered in CNS tumors. Consequently, we recommend searching for alternative alterations of the *PLAG1* gene in the event of a radiological and histopathological suspicion of this diagnosis when *PLAGL1/2* amplifications have not been found.

### Supplementary Information

Below is the link to the electronic supplementary material.

Supplementary file1: Fig. 1 Methylation-based t-SNE distribution. Reference DNA methylation classes (v12.5 of the DKFZ classifier): EFT_CIC: CIC-rearranged sarcoma; EPN_MPE: myxopapillary ependymoma; EPN_PFA: ependymoma, posterior fossa groupA; EPN_PFB: ependymoma, posterior fossa group B; EPN_ZFTA: ependymoma, ZFTA fusion; EPN_YAP: ependymoma, YAP fusion; HGNET_BCOR: central nervous system tumor with BCOR internal tandem duplication; HGNET_MN1: astroblastoma, MN1-altered; HGNET_PATZ: neuroepithelial tumor with PATZ1 fusion; HGNET_PLAG: embryonal tumor with PLAG-family amplification; NET_PLAGL1: neuroepithelial tumor with PLAGL1-fusion. (TIFF 235 KB)

Supplementary file2: Fig. 2 Schematic representation of PLAG1 fusions. The EWSR1 and TCF4 genes contribute no significant functional domain. The putative PLAG1 fusion proteins will contain 5 functional zinc finger domains out of the 7 in the wild-type protein. (TIF 65 KB)
