# Supplementary figures and images for: PLAG1 fusions extend the spectrum of PLAG(L)-altered CNS tumors
Source: Acta Neuropathol. 2023 Oct 23;146(6):841–4. doi: 10.1007/s00401-023-02643-4 (PMC10627894; doi:10.1007/s00401-023-02643-4)

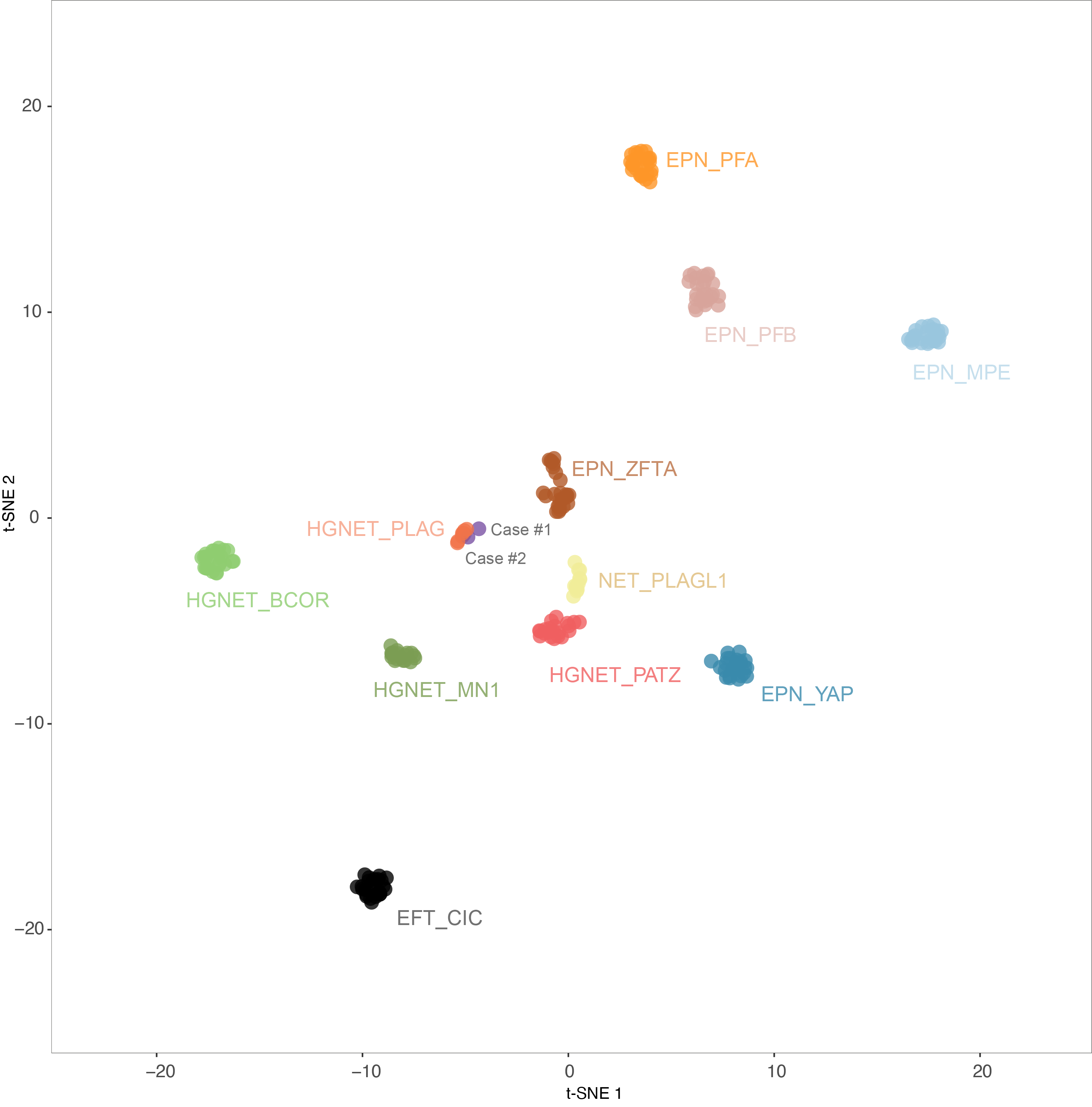

Supplement: Supplementary file 1 — Supplementary file1: Fig. 1 Methylation-based t-SNE distribution. Reference DNA methylation classes (v12.5 of the DKFZ classifier): EFT_CIC: CIC-rearranged sarcoma; EPN_MPE: myxopapillary ependymoma; EPN_PFA: ependymoma, posterior fossa groupA; EPN_PFB: ependymoma, posterior fossa group B; EPN_ZFTA: ependymoma, ZFTA fusion; EPN_YAP: ependymoma, YAP fusion; HGNET_BCOR: central nervous system tumor with BCOR internal tandem duplication; HGNET_MN1: astroblastoma, MN1-altered; HGNET_PATZ: neuroepithelial tumor with PATZ1 fusion; HGNET_PLAG: embryonal tumor with PLAG-family amplification; NET_PLAGL1: neuroepithelial tumor with PLAGL1-fusion. (TIFF 235 KB) [file 401_2023_2643_MOESM1_ESM.tiff]

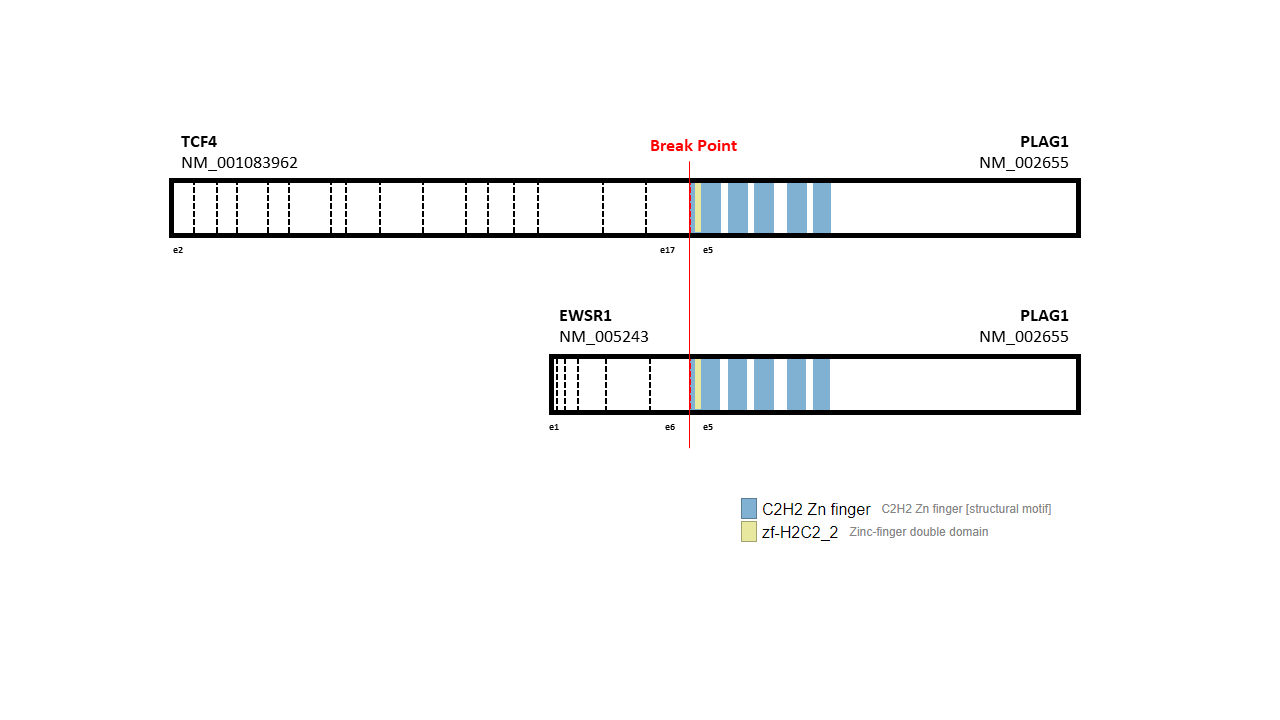

Supplement: Supplementary file 2 — Supplementary file2: Fig. 2 Schematic representation of PLAG1 fusions. The EWSR1 and TCF4 genes contribute no significant functional domain. The putative PLAG1 fusion proteins will contain 5 functional zinc finger domains out of the 7 in the wild-type protein. (TIF 65 KB) [file 401_2023_2643_MOESM2_ESM.tif]
